# Expression, Purification, and Identification of Associated Proteins of the Full-length hCDK12/CyclinK Complex*[Fn FN2]

**DOI:** 10.1074/jbc.M114.612226

**Published:** 2014-11-26

**Authors:** Bartlomiej Bartkowiak, Arno L. Greenleaf

**Affiliations:** From the Department of Biochemistry, Duke University Medical Center, Durham, North Carolina 27710

**Keywords:** C-terminal Domain (Carboxyl Tail Domain, CTD), Cyclin-dependent Kinase (CDK), Enzyme Purification, RNA Polymerase II, RNA Processing, Analog-sensitive Kinase, Baculovirus

## Abstract

The coupling of transcription and associated processes has been shown to be dependent on the RNA polymerase II (RNAPII) C-terminal repeat domain (CTD) and the phosphorylation of the heptad repeats of which it is composed (consensus sequence Y_1_S_2_P_3_T_4_S_5_P_6_S_7_). Two primary S_2_ position CTD kinases have been identified in higher eukaryotes: P-TEFb and CDK12/CyclinK. The more recently discovered CDK12 appears to act at the 3′-end of the transcription unit and has been identified as a tumor suppressor for ovarian cancer; however much is still unknown about the *in vivo* roles of CDK12/CyclinK. In an effort to further characterize these roles we have purified to near homogeneity and characterized, full-length, active, human CDK12/CyclinK, and identified hCDK12-associated proteins via mass spectrometry. We find that employing a “2A” peptide-linked multicistronic construct containing CDK12 and CyclinK results in the efficient production of active, recombinant enzyme in the baculovirus/Sf9 expression system. Using GST-CTD fusion protein substrates we find that CDK12/CyclinK prefers a substrate with unmodified repeats or one that mimics prephosphorylation at the S_7_ position of the CTD; also the enzyme is sensitive to the inhibitor flavopiridol at higher concentrations. Identification of CDK12-associating proteins reveals a strong enrichment for RNA-processing factors suggesting that CDK12 affects RNA processing events in two distinct ways: Indirectly through generating factor-binding phospho-epitopes on the CTD of elongating RNAPII and directly through binding to specific factors.

## Teaser

**Background:** CDK12 helps to regulate DNA transcription and associated processes.

**Results:** Full-length and active CDK12 can be expressed using baculovirus and CDK12 interacts with many RNA processing factors.

**Conclusion:** CDK12 may affect RNA processing events both directly and indirectly.

**Significance:** A better understanding of CDK12 activities will be useful in deciphering the nuances of transcription and in drug discovery.

## Introduction

Eukaryotic transcription and the attendant processing of pre-mRNA require a precise coordination between the transcription machinery and the recruitment and activity of transcription-associated factors. Many facets of this coupling between transcription and associated processes have been shown to be dependent on a particular feature of RNA polymerase II (RNAPII),^2^ the C-terminal repeat domain or CTD (1). An extension of the polymerase's largest subunit, Rpb1, the CTD is composed of a tandem array of seven amino acid repeats with the consensus sequence Y_1_S_2_P_3_T_4_S_5_P_6_S_7_. The number of these repeats varies depending on the organism and appears to correlate with genomic complexity (there are 26 repeats in yeast, 42–45 in *Drosophila*, and 52 in mammals), which makes intuitive sense given the CTD function as a selective and flexible binding scaffold for transcription associated factors. The CTD has been implicated in many transcription related phenomena and has been shown to be especially important for mRNA maturation (5′-end capping, splicing, 3′-end processing, and nuclear export) and chromatin modification (1–4).

The binding specificity of the CTD is chiefly determined by its phosphorylation state, which undergoes a series of alterations throughout the transcription cycle. In a highly simplified model initiated and early transcribing polymerases are phosphorylated at the Ser-5 and Ser-7 positions of the heptad repeats while actively elongating polymerases progressively gain phosphates at the Ser-2 and lose them at the Ser-5 positions; at the 3′-end of the transcription unit Ser-2 phosphorylation appears to predominate at the expense of the other modifications (2, 3). The CTD phosphorylation state is in turn regulated through the activity of various phosphatases and the transcription-regulating cyclin-dependent kinases (CDKs): In humans these transcriptional CDKs are CDK7, CDK8, CDK9, CDK12, and possibly CDK13 (5, 6). Much akin to their cell cycle regulating counterparts, the transcriptional CDKs activity depends on their interaction with their regulatory cyclin partners and on activating posttranslational modifications; but in contrast the levels of the transcriptional cyclin proteins do not fluctuate in concert with the cell cycle.

**FIGURE 1.**
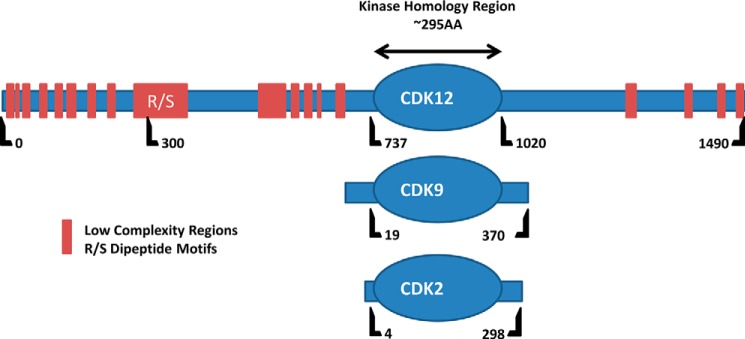
**Comparison of hCDK12 to other CDKs.** Schematics of CDK12, CDK9, and CDK2 primary structure, relative amino acid positions are indicated below each sequence with the kinase homology domain highlighted as an *oval*. Unlike most CDKs, CDK12 contains long N- and C-terminal arms that extend from the Ser/Thr kinase homology domain. These arms contain stretches of low complexity (LC) sequence (marked in *red*) and RS domains; motifs characteristic of splicing factors and believed to mediate protein-protein interactions.

The majority of Ser2 CTD phosphorylation on productively elongating RNAPII in yeast (*Saccharomyces cerevisiae*) appears to be catalyzed by CTDK-I, a three subunit enzyme consisting of Ctk1 (a CDK homolog), Ctk2 (a cyclin homolog), and Ctk3 (function unknown). Ctk1 is not the only Ser-2 CTD kinase in yeast, the essential Bur1 kinase (Bur1 is a CDK homolog and its cyclin partner is Bur2) has also been shown to contribute to Ser-2 phosphorylation. Likewise two primary Ser-2 position CTD kinases have been identified in higher eukaryotes: The Bur1 related P-TEFb (composed of CDK9 and cyclinT) and the CTDK-I related CDK12/CyclinK complex. P-TEFb is recruited near the 5′-end of the transcription unit and in addition to Ser-2 of the CTD it is able to phosphorylate NELF and the elongation factor Spt5, making it essential for entry into productive transcriptional elongation. The more recently discovered CDK12/CyclinK on the other hand appears to act further downstream within the transcription unit and currently its best studied phosphorylation target is the Ser-2 position of the CTD (7, 8). Interestingly, human CDK12 (hCDK12) is uncharacteristically large with a molecular weight of ∼160 kDa (most CDKs are about 50 kDa) and contains large N- and C-terminal extensions that flank the CDK kinase domain (Fig. 1). These extensions contain multiple stretches of low complexity sequence and RS domains (domains characteristic of splicing factors and regulators believed to mediate protein-protein interactions (9)); as expected of RS domain containing proteins, hCDK12 is found in nuclear speckles (believed to be storage sites for splicing factors, Ref. 9) (10).

Recent studies have elucidated the structure of a truncated form of the CDK12/CyclinK complex *in vitro* and implicated CDK12/CyclinK in the 3′-end processing of the *MYC* gene *in vivo* (11, 12). In addition hCDK12 has been identified as a tumor suppressor for ovarian cancer (13); however much is still unknown about the *in vivo* roles of CDK12/CyclinK. In an effort to further characterize these roles we have purified to near homogeneity and characterized full-length, active, human CDK12/CyclinK and identified hCDK12-associated proteins via mass spectrometry.

## EXPERIMENTAL PROCEDURES

### 

#### 

##### Antibodies and Western Blot Analysis

The anti-hCDK12 antibody consists of rabbit affinity-purified IgGs directed against a peptide comprising amino acids 201–220 of hCDK12 (NCBI RefSeq: NP_057591.2). Anti-CyclinK antibody was purchased from Abcam (ab130475). Western blot analysis was performed using the Odyssey infrared scanner and secondary antibodies from Li-Cor.

##### GST-CTD Substrates

GST-yeastCTD (GSTy-CTD) was purified as previously described (14). Mutant GST-CTD substrate constructs (WT, S2A, S2E, S5A, and S5E (originally designed by Jeff Corden's laboratory (15)) were kindly provided by Dr. Aseem Ansari's laboratory. The S7E construct was created by gene synthesis of DNA containing 16 CTD heptad repeats with each Ser-7 position replaced with Glu, followed by an HA tag (YPYDVPDYA), a single glutamate, and a stop codon (to be identical to the WT GST-CTD substrate originally designed by the Corden Laboratory). The sequence also incorporates a BamHI and SalI site at the 5′- and 3′-end, respectively (IDT). The S7E CTD sequence was cloned into the pGEX-5X1 vector (GE Healthcare: Life Sciences) using the BamHI and SalI restriction sites. Expression of all constructs was performed in BL21-Codon Plus (DE3)-RIPL cells (expression conditions were 25 °C with 1 mm IPTG; Stratagene). After 16 h of expression, cells were sonicated, and the fusion protein was purified using a glutathione-agarose (Pierce) column following the manufacturer's protocol. Peak elution fractions were then pooled and submitted to size exclusion chromatography (Superdex 200 HR 10/30, Amersham Biosciences). Peak size exclusion fractions were pooled and buffer exchanged (Amicon Ultra Centrifugal Filters) into 25 mm HEPES pH 7.6 with 50 mm NaCl to a final protein concentration of 0.5 μg/μl.

##### CTD Kinase Assays

CTD kinase assays were performed essentially as described previously (16), with minor variations: Kinase reactions included either 30 or 300 μm ATP, 10 μCi of [γ^32^-P]ATP, 5 mm MgCl_2_, 25 mm Tris, pH 7.6, 5% glycerol, 2 mm DTT, 150 mm NaCl, 1 μg of GST-yCTD, and 50–100 ng of hCDK12/CyclinK in a 25 μl reaction volume. Reactions were performed for 15–45 min (specified in each figure) at 37 °C. Reaction times and enzyme amounts were selected to be in the range where phosphate incorporation is linear with time. Phosphorylated products were visualized using film (Kodak Biomax MR) or a PhosphorImager; quantification was performed using a PhosphorImager and ImageQuant software. Ess1 was provided by Dr. Pei Zhou's laboratory. 1-NM-PP1 was purchased from Axon Medchem and dissolved in DMSO. For DRB and flavopiridol (FVP) inhibition, equal activity of CDK9, provided by the laboratory of Dr. David Price, and CDK12/CyclinK was used (0.6 μm final concentration of CDK9/CyclinT in the reaction mixture). FVP was purchased from Sigma Aldrich (F3055) and DRB was provided by Dr. Shudong Wang from the University of Nottingham. Inhibitors were dissolved in DMSO and then aliquoted at the appropriate concentrations in 4% DMSO. 1 μl of either 4% DMSO or the appropriate concentration of each inhibitor in 4% DMSO was added to the reaction mixture for a final volume of 25 μl. Analysis of the inhibition data were performed using GraphPad Prism 6.

##### Construction of CDK12/CyclinK Expression Constructs and Mutant

The cDNA for hCDK12 was purchased from Open Biosystems (Clone ID: 9021722; NCBI accession BC140854), as was the cDNA for the short, 40 kDa, form of hCyclinK (Clone ID: 3907416; NCBI accession BC015935). The full-length hCyclinK cDNA was a very kind gift from Dr. Grace Cheng and Dr. Gregg Morin (17).

The hCDK12 cDNA was then extended using PCR: The forward primer contained an upstream NarI extension (GTAGCAGGCGCCATGCCCAATTCAGAGAGACA) and the reverse primer annealed just upstream of the hCDK12 stop codon, replacing it with an extension containing a part of the P2A sequence with a BamHI restriction site (TTGCTTTAACAGAGAGAAGTTCGTGGCTCCGGATCCGTAAGGAACTCCTCTCCCTCTTC).

The hCyclinK cDNAs were also extended using PCR: The forward primer contained part of the P2A sequence (CAAGCAGGAGACGTGGAAGAAAACCCCGGTCCTATGAAGGAGAATAAAGAAAATTCAAGC) and the reverse primer contained an extension containing a NotI restriction site (GTAGCAGCGGCCGCTTATCTCATCCAGGCTGCCC). This extended hCyclinK PCR product was then extended again using the reverse primer from above and another forward primer which added more of the P2A sequence, including the aforementioned BamHI restriction site (GGATCCGGAGCCACGAACTTCTCTCTGTTAAAGCAAGCAGGAGACGTGGAAGA).

The hCDK12 PCR product was then cloned into the pFastBac HT B (Invitrogen) vector using NarI and BamHI restriction sites. The resulting plasmid was further modified by the addition of the full-length or 40 kDa, fully extended, hCyclinK PCR products using the BamHI and NotI restriction sites; the resulting plasmids were the final pFastBac HT B hCDK12/CyclinK and the pFastBac HT B hCDK12/CyclinK (40kDa) expression constructs.

Mutations (D877N, K756A, and F813G) were introduced into the pFastBac HT B hCDK12/CyclinK expression construct using the QuikChange site-directed mutagenesis kit (Stratagene).

##### Expression and Purification of hCDK12

Expression was performed essentially as described in the Bac-to-Bac Baculovirus Expression System instruction manual (Invitrogen). Miniprepped pFastBac HT B hCDK12/CyclinK plasmids were transformed into DH10Bac *Escherichia coli* for transposition into the expression bacmid. Transformed colonies were screened for the CDK12/CyclinK insert by PCR using M13 primers and a primer internal to hCDK12 (GACTGACCGACTGCCTTCTC). Bacmids were prepared from 150 ml DH10Bac cultures with the NucleoBond Xtra Midi kit (Macherey-Nagel) and transfected into *Spodoptera frugiperda* (Sf9) insect cells using CellfectinII (Invitrogen) following the manufacturer's protocol. Sf9 cells were maintained in Hyclone SFX-Insect Media (Thermo Scientific) at 27 °C.

Virus amplification was performed as described in the Bac-to-Bac manual for 3 rounds of amplification. Viral titer was determined via Western blotting of the cell culture supernatant with the anti-gp64 antibody against baculovirus envelope protein (eBioscience). Expression was optimized by varying the amount of virus (using Western blotting for gp64 for relative quantification) and time of expression; expression was monitored through Western blotting for hCDK12. For purification, 500 ml of Sf9 cells at 1 × 10^6^ cells/ml were infected with baculovirus, incubated for 96 h, and cells were collected by centrifugation at 730 × *g* for 15 min at 4 °C (all subsequent steps at 4 °C), washed with ice cold 1× PBS, and pelleted again.

Cells were resuspended in cytoplasmic lysis buffer (10 mm HEPES, pH 8.0, 320 mm sucrose, 3 mm calcium chloride, 2 mm magnesium acetate, 1 mm DTT, 5 mm NaF, and 0.5% Nonidet P-40) with protease inhibitor mixture for tissue extracts (P8340, Sigma). The resulting slurry was homogenized using 20 strokes with a tight fitting pestle of a dounce homogenizer. Nuclei were then pelleted by centrifugation at 1,500 × *g* for 15 min. The supernatant was removed, and the nuclei were resuspended in 6 ml of nuclear resuspension buffer (20 mm HEPES, pH 8.0, 1.5 mm MgCl_2_, 20 mm KCl, 25% glycerol, 1 mm DTT, 5 mm NaF, and protease inhibitor mixture for tissue extracts). Nuclei were then lysed by the dropwise addition (with stirring) of 6 ml of nuclear extraction buffer (80 mm HEPES, pH 7.6, 1.2 m NaCl, 20% glycerol, 0.06% Triton X-100, 1 mm DTT, 5 mm NaF, and protease inhibitor mixture for tissue extracts). 6 ml of PEG solution (18% PEG 8000, 1 m NaCl, 1 mm DTT, 5 mm NaF, and protease inhibitor mixture) was then added, and the solution was incubated at 4 °C for 45 min with rotation. The insoluble fraction was removed by centrifugation at 20,000 × *g* for 30 min. The soluble lysate containing hCDK12/CyclinK was then adjusted to 20 mm imidazole and bound in bulk to a preequilibrated Ni-Sepharose 6 Fast Flow column (GE Healthcare) for 2.5 h at 4 °C rocking end over end. The resin was collected and washed with 10 column volumes of high salt wash buffer (50 mm Tris-HCl, pH 8.0, 500 mm NaCl, and 20 mm imidazole). The protein was eluted with 50 mm Tris-HCl, pH 8.0, 300 mm NaCl, 250 mm imidazole, 1 mm DTT, 5 mm NaF, 8% glycerol, and protease inhibitor mixture. The elution peak from the Ni-Sepharose was pooled and, using a 500 μl loading loop, applied to an ÄKTA FPLC system equipped with a Superdex 200 10/30 HR column (GE Healthcare) at a flow rate of 0.25 ml/min with 25 mm HEPES pH 7.6, 8% glycerol, and 300 mm NaCl as the running buffer. 500-μl fractions were collected, and fractions containing hCDK12/CyclinK were identified by SDS-PAGE and flash frozen for storage at −80 °C until use.

##### Phosphatase Treatment

20 μl of a CDK12/CyclinK Ni-Sepharose elution fraction was treated with 10 units of FastAP Thermosensitive Alkaline Phosphatase (Thermo Scientific) at 37 °C for 1 h. The reaction was stopped by the addition of 5× SDS loading buffer (Laemmli buffer) and analyzed by SDS-PAGE.

##### Immunoprecipitation for Mass Spectrometry

Dignam-Roeder nuclear extracts (18) (protein concentration at ∼16 mg/ml) of HeLa cells were kindly provided by Dr. Irina Evsyukova from Dr. Mariano Garcia-Blanco's laboratory. The nuclear extracts were diluted to ∼4 mg/ml total protein, and the salt concentration was adjusted to 400 mm NaCl. (All steps are performed at 4 °C with protease inhibitors (Sigma P8340) and 1 mm DTT present in all buffers.) The dilute solution was cleared via centrifugation (20,000 × *g* at 4 °C for 30 min), and 2 ml was used for each pulldown.

SoftLink Soft Release Avidin Resin (Promega), which was selected due to its low background binding, was saturated with a biotinylated goat anti-Rabbit IgG antibody (Jackson ImmunoResearch Laboratories, Code 111-065-008). 10 μl of the resulting anti-Rabbit IgG resin was used to preclear each 2-ml aliquot of the diluted HeLa nuclear extract (1 h of end over end rocking, 4 °C). The rest of the anti-rabbit IgG resin was either saturated with the rabbit anti-hCDK12 antibody or with total rabbit IgGs (purified using protein A-agarose from rabbit pre-immune serum) as a negative control; 20 μg of antibody was used per 10 μl of the anti-rabbit IgG resin. The resulting bead-antibody-antibody complexes were then crosslinked using dimethyl pimelimidate (DMP) as described in Antibodies: A Laboratory Manual (19). Following crosslinking, the beads were rinsed with 100 mm formic acid and thoroughly washed with 1× PBS, 0.02% Tween 20. The crosslinked anti-CDK12 and total IgG beads were then used for overnight pulldowns using the precleared HeLa nuclear extracts as input (10 μl of the resin was used per 2 ml of nuclear extract).

Following the immunoprecipitation each 10 μl set of beads is washed 5 × 1 ml of IP buffer (Dignam-Roeder buffer D supplemented to 400 mm NaCl, see Ref. 18), 1 × 1 ml of 1× PBS with 1%Nonidet P-40, 3 × 1 ml 1× PBS 0.02% Tween 20, and 5 × 1 ml 1× PBS. The beads were spun down, supernatant was completely removed, and the beads were eluted with 100 mm formic acid (100 μl of 100 mm formic acid per 10 μl of beads, 5 min, room temperature, with agitation). The supernatant was then removed, and 200 μl of each sample (total rabbit IgG control and anti-hCDK12) was submitted to the Duke Proteomics Facility for lyophilization and subsequent analysis (LC MS/MS).

## RESULTS

### 

#### 

##### Construction of the hCDK12 Expression Construct

**FIGURE 2.**
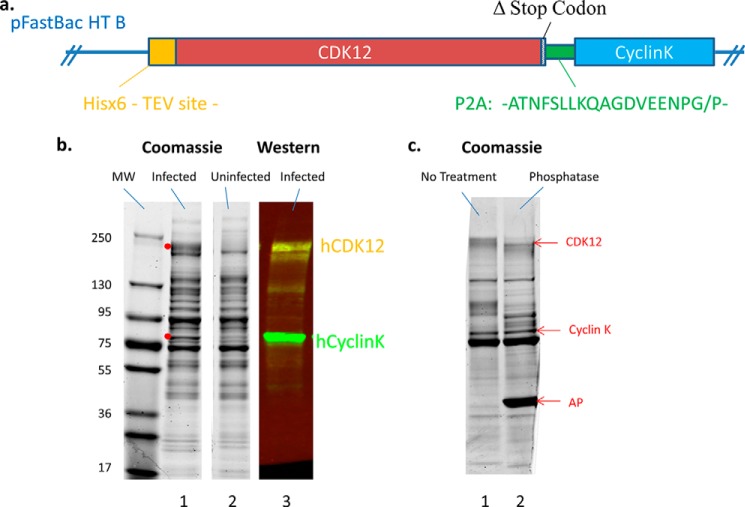
**The hCDK12/CyclinK baculovirus construct and its expression.** *a*, schematic of the hCDK12/CyclinK expression construct which consists of the full-length human CDK12 gene (lacking its stop codon) fused to the P2A sequence from porcine teschovirus and followed by the full-length sequence for hCyclinK. The complete construct was cloned into the MCS of the pFastBac HT B insect cell expression vector (Invitrogen), which led to the inclusion of a 6× His tag followed by a TEV protease site at the N-terminal end of hCDK12. *b*, Coomassie-stained gels and Western blots of Ni column elution fractions of hCDK12/CyclinK baculovirus infected and uninfected Sf9 cell culture lysates. Infection of Sf9 cells with hCDK12/CyclinK baculovirus results in the appearance of two additional bands on the Coomassie-stained elution fractions (*red dots*). Western blot analysis of infected elution fractions with anti-hCDK12 (in *yellow*) and anti-hCyclinK (in *green*) antibodies indicate the presence of soluble hCDK12/CyclinK complex in the infected lysates. *c*, Coomassie-stained gel of Ni column fractions of hCDK12/CyclinK baculovirus-infected Sf9 cell lysates untreated and treated with alkaline phosphatase (AP).

It has been previously reported in the literature that full-length CDK12 expressed in baculovirus-infected cells is highly insoluble or inactive (10, 11). We hypothesized that the reason for this may be the lack of appropriate levels of CDK12 cyclin partner, which would be likely to affect the stability of the kinase. To address this problem we employed a “2A” peptide-linked multicistronic construct (20), allowing for the expression of CDK12 and CyclinK in a one to one ratio from a single open reading frame. The final construct contained the His-tagged hCDK12 transcript variant 2 sequence (GenBank^TM^ accession NM_015083) lacking the final stop codon (1481AA; transcript variant 1 (Genbank^TM^ accession NM_016507) contains an extra 9 AAs from exon 14, which map to the C-terminal arm of the kinase), the 2A sequence from porcine teschovirus-1 (19AA), and the full sequence for CyclinK (580AA) (Fig. 2*a*) (17). Co-expression of both the CDK and its cyclin partner in baculovirus-infected Sf9 cells followed by Ni column purification resulted in the appearance of two new bands in Coomassie gels of the elution fractions; a diffuse band at ∼180 kDa and a sharp band at ∼75 kDa. These bands were confirmed to be CDK12 and CyclinK by Western blotting (Fig. 2*b*). The diffuse nature of the CDK12 band is likely due to a high degree of post-translational modification; phosphorylation of CDK12 has been reported previously (10) and phosphatase treatment of Ni column elutions of CDK12 resulted in a mobility shift and partial collapse of the CDK12 Coomassie band (Fig. 2*c*). Therefore co-expression of CDK12 with its cyclin partner alleviates solubility issues and is a viable strategy for the production of recombinant enzyme.

##### Kinase Assays of WT hCDK12, Kinase Dead, and Analog-sensitive Mutants

**FIGURE 3.**
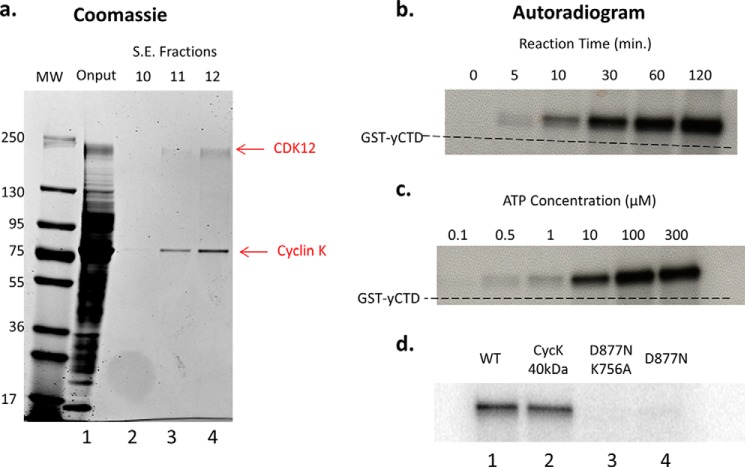
**Purification and CTD kinase activity of hCDK12/CyclinK complex and mutants.** *a*, Coomassie-stained gel of size exclusion chromatography (Superdex 200 HR 10/30) fractions from a hCDK12/CyclinK purification. Input (*lane 1*) consists of pooled Ni column elution fractions of hCDK12/CyclinK baculovirus-infected Sf9 cell nuclear extracts. The majority of fraction 10 (*lane 2*) consists of column void volume while fraction 11 (*lane 3*) is the first true elution fraction. *b*, purified hCDK12/CyclinK CTD kinase activity was assayed in the presence of [γ^32^-P]ATP using 1 μg of a GST-yCTD fusion protein (26 heptad repeats) as substrate and reaction mixtures were analyzed by SDS-PAGE. Autoradiograms of the SDS-PAGE gels revealed a time (ATP at 300 μm) and *c*, ATP-dependent (reaction time at 45 min) CTD kinase activity. The SDS-PAGE mobility of the unphosphorylated/unshifted GST-yCTD substrate is indicated via a *dashed line. d*, autoradiograms of CTD kinase assays using purified mutants of the hCDK12/CyclinK complex. Equal amounts of each complex are used in the reactions; hCDK12/CyclinK complex containing a shortened form of CyclinK exhibited comparable activity to full-length wild-type complex (compare *lanes 1* and *2*), while a kinase dead double (*lane 3*) and single mutant (*lane 4*) exhibited little to no residual activity.

**FIGURE 4.**
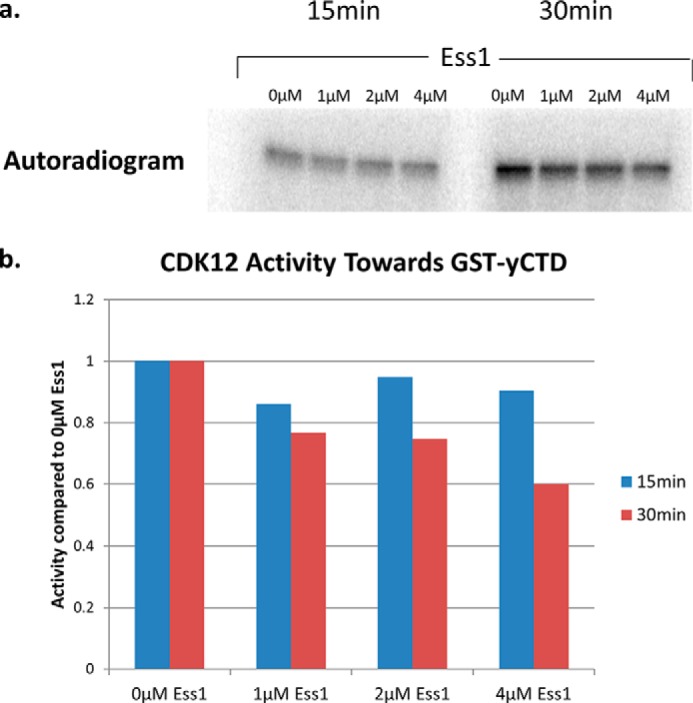
**The effect of a prolyl isomerase on the activity of hCDK12.** *a*, an autoradiogram of a SDS-PAGE gel from an hCDK12/CyclinK kinase assay with increasing amounts of the prolyl isomerase Ess1. Assays were performed using 1 μg of GST-yCTD as a substrate at 30 μm ATP, 37 °C, and 15/30 min reaction times. *b*, quantification of the degree of phosphorylation of each mutant substrate using a PhosphorImager and ImageQuant software (normalized to a value of 1.0 for the 0 μm Ess1). No stimulation of hCDK12 activity was observed.

His-tagged CDK12 was purified from baculovirus-infected cells via isolation of nuclei, followed by nickel column chromatography and size exclusion chromatography (see “Experimental Procedures”). Serendipitously the large size of the CDK12/cyclinK complex, coupled with the high likelihood of non-globular structural elements in the N- and C-terminal arms of its components, led it to elute near the void volume of the size exclusion column. Therefore a simple two-step purification strategy yielded highly purified complex which could be employed in subsequent studies (Fig. 3*a*). Purified CDK12/CyclinK exhibited a time and ATP-dependent CTD kinase activity when assayed using GST-yeastCTD (GST-yCTD) fusion proteins as substrate in the presence of [γ^32^-P]ATP (Fig. 3, *b* and *c*). Unlike CTDK-I, which is inhibited by the presence of additional salt in the kinase buffer, CDK12/CyclinK activity was stimulated by salt (NaCl and KCl were tested at 0, 150, and 200 mm) and strongly inhibited by detergents (0.05% Triton X-100, Nonidet P-40, and Tween 20 were tested) (21) (data not shown). We also tested hCDK12/CyclinK activity toward the GST-yeastCTD in the presence of the yeast proline isomerase Ess1, but observed no stimulation of activity (Fig. 4). In addition to the full length construct, we also constructed and purified a CDK12/CyclinK complex containing the originally identified 40-kDa form of CyclinK (7, 8, 17, 22); however we could not detect any difference in GST-yeastCTD kinase activity between the full length and shortened CDK12/CyclinK complex (Fig. 3*d*).

In the interest of creating a negative control for our assays we also set out to generate a kinase dead mutant of CDK12. Based on the literature we focused our attention on mutating the conserved aspartate residue known to be essential for the phospho-transfer reaction in other CDKs (Ex: D145 of CDK2 and D167 of CDK9) (23, 24); alignment of the sequences of several CDKs identified the most probable candidate residue to be aspartate 877 of CDK12. Mutation of this residue to asparagine drastically reduced kinase activity to almost background levels, although some residual activity was observed on longer exposures and with high amounts of enzyme (Fig. 3*d* and data not shown). Following up on this result, we eliminated the possibility of a copurifying CTD kinase activity by purifying and assaying the D867N mutant using antibody-saturated beads (size exclusion fractions were used as input and beads were subsequently washed with 1 m NaCl and 0.5% Nonidet P-40); however the slight residual activity persisted. In view of this, we made an additional mutation in the nucleotide binding site of CDK12: K756A modeled on the CDK2 K33A mutation (25). The double mutant kinase had even less background activity, although some slight residual activity could still be detected at extremes of exposure and enzyme amounts (Fig. 3*d*). Despite this slight residual activity, compared with wild-type kinase, for most intents and purposes both the single and double mutants can be considered to be inactive (also see Ref. 13). Accordingly, mutation of D877N and D877N/K756A inhibits the vast majority of kinase activity and confirms the purity of our CDK12/CyclinK purification.

**FIGURE 5.**
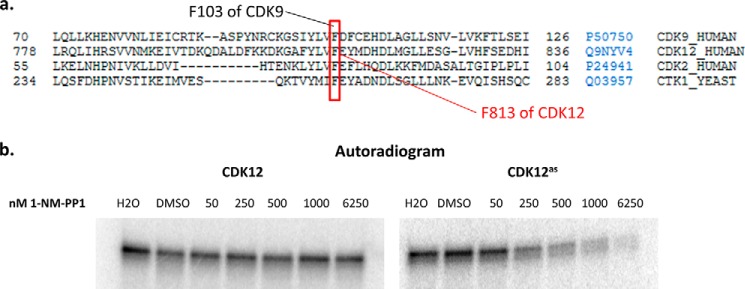
**Developing an analog-sensitive mutant of CDK12.** *a*, sequence alignment (UniProt) of CDKs (yeast Ctk1 and human CDK2, CDK9, and CDK12) identifying the potential “gatekeeper” residue of hCDK12. Relative amino acid positions are indicated at the beginning and end of each sequence and each protein's UniProt accession number and name are displayed on the right. Conserved phenylalanine residues that have been previously mutated to make analog-sensitive kinases are highlighted with a *red box. b*, purified hCDK12/CyclinK (CDK12) and F813G hCDK12/CyclinK mutant (CDK12^as^) CTD kinase activity was assayed using 1 μg of GST-yCTD fusion protein at 30 μm ATP, 37 °C, and a 30 min reaction time with increasing concentrations of the bulky adenine analog 1-NM-PP1 in DMSO; the reactions were analyzed by SDS-PAGE and visualized using a PhosphorImager.

To inform future experiments we also engineered an analog sensitive mutant of CDK12. Analog-sensitive kinases are created by mutating a large phenylalanine residue, termed the “gatekeeper” residue, near the enzyme active site to a much smaller glycine; the mutated enzyme is then able to accept bulky adenine analogs in its active site, allowing for specific inhibition of its activity (27, 28). Alignment of CDK12 with other CDKs and comparison to other analog-sensitive kinases (29) implicated phenylalanine 813 of CDK12 as a candidate “gatekeeper” residue (Fig. 5*a*); this residue was mutated to glycine to create CDK12^as^. Purified CDK12^as^/CyclinK kinase activity was assayed alongside wild-type kinase in the presence of increasing amounts of the bulky adenine analog 1-NM-PP1. Addition of 1-NM-PP1 had no effect on the activity of the wild-type kinase in the GST-yCTD kinase assay; however addition of 1-NM-PP1 to reactions containing CDK12^as^ resulted in a virtually complete loss of enzyme activity (Fig. 5*b*). Consequently the two step purification process results in highly purified CDK12/CyclinK devoid of contaminating kinase activities. In addition the high sensitivity of CDK12^as^ to 1-NM-PP1 suggests that it will be a useful tool for future studies of CDK12 kinase activity *in vivo*.

##### CDK12 Substrate Specificity and Activity in the Presence of DRB and FVP

**FIGURE 6.**
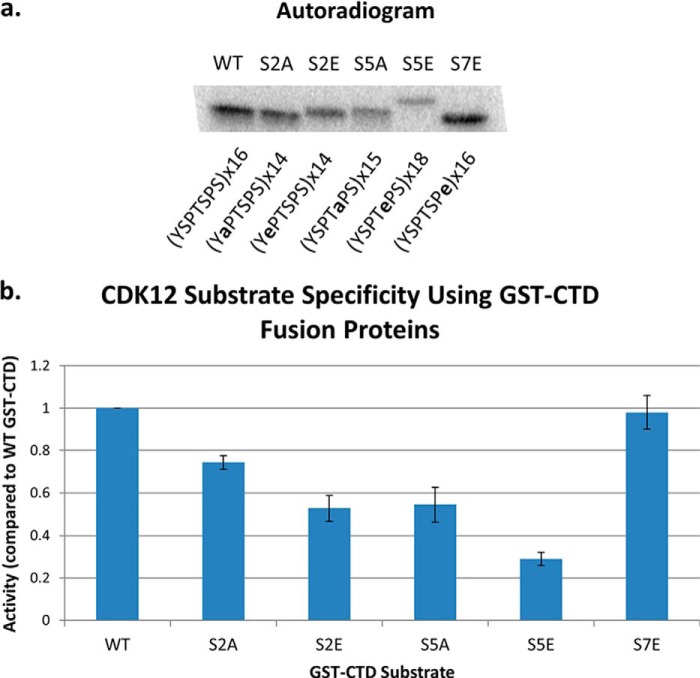
**Phosphorylation of mutant GST-CTD substrates by hCDK12/CyclinK.** *a*, representative autoradiogram of a SDS-PAGE gel from an hCDK12/CyclinK kinase assay using mutant GST-CTD substrates. The composition and number of the CTD heptad repeats in the GST-CTD fusion protein is indicated below the appropriate lanes. *b*, quantification of the degree of phosphorylation of each mutant substrate using a PhosphorImager and ImageQuant software (normalized to a value of 1.0 for the wild-type GST-CTD fusion protein). Error bars are ± 2 S.E., *n* = 3.

Using a series of synthetic three heptad repeat phospho-peptides and truncated forms of the hCDK12/CyclinK complex, previous studies have reported that hCDK12 exhibits a marked preference toward a CTD phosphorylated at the Ser-7 position within the heptad repeat (11). To investigate the substrate specificity of the full-length hCDK12/CyclinK complex toward a longer CTD substrate we used bacterially expressed GST-CTD fusion proteins carrying ∼15 wild-type or Ser-substituted repeats: WT (YSPTSPS), S2A (YAPTSPS), S2E, S5A, S5E, and S7E. These constructs either eliminate a potential phosphoacceptor (serine to alanine) or act as a phosphomimetic (serine to glutamate, which resembles phospho-serine). Under our standard kinase assay conditions (300 μm ATP, 1 μg GST-CTD fusion protein substrate, 30 min reaction time) we find that hCDK12/CyclinK does exhibit a preference toward the S7E GST-CTD construct when compared with the other mutant CTD constructs, but not when compared with wild-type CTD construct which was phosphorylated to a similar extent (Fig. 6, *a* and *b*). Kinase activity is significantly weaker toward the S2E and S5A substrates (∼50% as compared with WT) with the S5E presenting itself as the worst substrate (∼30% activity as compared with WT). The S2A construct was also a somewhat weaker substrate than WT or S7E (∼75% activity as compared with WT) (Fig. 6, *a* and *b*).

**FIGURE 7.**
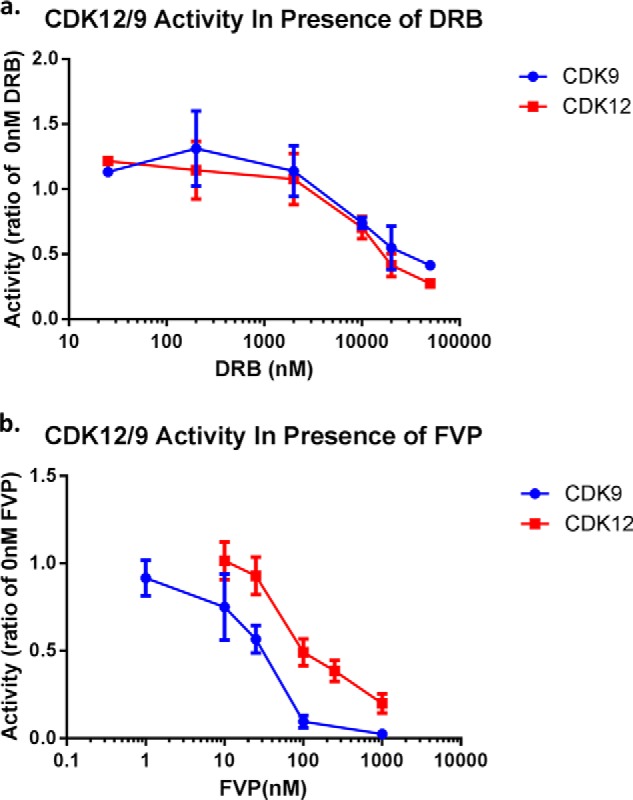
**CDK9 and CDK12 activity in the presence of DRB and FVP.** Concentration series of (*a*) DRB and (*b*) FVP for CDK12/CycK and CDK9/CycT1 activity assayed using 1 μg of GST-yCTD (30 μm ATP, 37 °C, 30-min reaction time). Activity of each kinase is normalized to a value of one for 0 nm DRB or 0 nm FVP, respectively. Error bars are ± 1 S.E. of the mean, *n* = 3 (except for DRB 25 nm and 5000 nm where *n* = 1 and 2, respectively).

Many studies of CDK9's role in RNAPII transcription and CTD phosphorylation employed the CDK9 inhibitor flavopiridol (FVP) (30). FVP has been shown to exhibit significant selectivity for CDK9 as compared with the other transcriptional CDKs (IC50 of 3–20 nm as compared with ∼300 nm for CDK7) and because of this presumed selectivity, the effects of FVP treatment have often been interpreted to be due to inhibition of CDK9 alone. However several other CDKs (CDK1, 2, 4, and 6) are highly sensitive to FVP (IC50s at ∼40 nm) (31) and experiments using a dominant negative form of CDK9 have suggested that the potent effects of FVP on transcription may be due to the inhibition of additional CTD kinases other than FVP (32). Therefore, we set out to determine if full length CDK12 also exhibited sensitivity to FVP. The binding of FVP to CDK9 is characterized by a very strong, long lived, hydrophobic interaction, resulting in a one to one binding of inhibitor to kinase (even at nanomolar concentrations of inhibitor) (30, 33). We decided to leverage this non-canonical inhibitor-kinase interaction, coupled with the more general behavior of the competitive CDK inhibitor 5,6-dichlorobenzimidazole riboside (DRB) to determine if CDK12 exhibits a similar susceptibility to inhibition by FVP. Assayed using GST-yCTD, under the same buffer conditions and activity, both CDK12 and CDK9 responded in the same way to increasing concentrations of the competitive inhibitor DRB (Fig. 7*a*). However, the behavior of CDK12 and CDK9 diverged when treated with FVP (Fig. 7*b*): Under the conditions used, CDK9 exhibited an IC50 of ∼25 nm (95% confidence interval of 18–37.6 nm) *versus* an IC50 of ∼150 nm for CDK12 (95% confidence interval of 104.6–208.2). This suggests that the inhibition of CDK12 by FVP does not result in the tight interaction mode exhibited by FVP and CDK9. Despite this difference, the activity of CDK12 is clearly inhibited by the higher concentrations of FVP; therefore whether CDK12 is inhibited by FVP *in vivo* will depend on intracellular inhibitor and ATP concentrations and remains an open question.

##### Identification of hCDK12-associated Proteins via Mass Spectrometry

As mentioned in the introduction, yeast CTDK-I functions as a heterotrimer composed of Ctk1, Ctk2, and Ctk3. Although previous studies have confirmed CDK12 cyclin partner to be cyclinK (7, 8, 17), as of the writing of this report, no other CDK12-associated proteins have been identified. To identify interacting proteins we immunopurified CDK12 from Dignam Roeder nuclear extracts of HeLa cells (18) using an affinity-purified anti-CDK12 antibody tethered to streptavidin beads. Immunopurified complexes were eluted and analyzed by mass spectrometry (LC MS/MS; Duke Proteomics Facility). Filtering out proteins present in control immunoprecipitations (immunoprecipitations using total rabbit IgG instead of anti-CDK12 IgG) results in a total of 309 identified proteins in the final hit list (supplemental File S1). Functional annotation of the positive hits using the DAVID database (34, 35) reveals a strong enrichment of mRNA splicing factors, mRNA-binding proteins, and nuclear speckle components. In agreement with this functional annotation, the presence of RS domains in the CDK12 N terminus, and the CDK12 localization to nuclear speckles (8, 10) we find serine/arginine repetitive matrix protein 1 and 2, and many SR Splicing Factors (SRSF1, 3, 4, 5, 6, 7, 8, 9, and 10) coprecipitating with CDK12. In addition, the mass spectrometry identified several other proteins of interest including PCF11 (a 3′-end processing factor) and components of the exon-junction complex (eIF4A3, MGN, RNPS1, and RBM8; previously shown to interact with SR proteins, Ref. 36). Although this initial list of CDK12 associated proteins will require further validation, it affirms an interaction between CDK12 and other RS domain containing proteins and reinforces the role of CDK12 as an important actor in cotranscriptional mRNA processing. It also raises the possibility that CDK12 affects RNA processing events in two distinct ways: indirectly through generating factor-binding phospho-epitopes on the CTD of elongating RNAPII, and directly through binding to specific factors via its extended N- and C-terminal domains.

## DISCUSSION

We have purified to near homogeneity full-length, active, human CDK12/CyclinK, the RNAPII CTD kinase orthologous to yeast Ctk1/2/3. It is highly likely that the key to heterologous expression of the full-length CDK12 protein in the baculovirus/Sf9 expression system is the one to one expression of both the kinase and its cyclin partner. Here we used a “2A” peptide-linked multicistronic construct in order co-express both hCDK12/CyclinK subunits and overcome previously reported solubility and post-translational modification problems. The overexpressed enzyme is heavily post-translationally modified, definitely by phosphorylation (probably on its N- and C-terminal extensions), but also through other modifications as phosphatase treatment does not completely resolve the diffuse nature of the hCDK12 band in SDS-PAGE gels. Although we do not know the exact degree of activating T-loop phosphorylation of the recombinant enzyme complex, the purified enzyme clearly exhibits CTD kinase activity toward an unmodified substrate (GST-yCTD); it would be interesting to see if coexpression of a CDK-activating kinase (such as yeast CAK) further enhanced this activity. Fortuitously, due to its uncharacteristically large size the purification of the recombinant His-tagged hCDK12/CyclinK protein can be performed from nuclear extracts with a straightforward two step procedure yielding highly purified complex.

Enzymatic characterizations of the purified kinase *in vitro* reveal features important to understanding CTD phosphorylation *in vivo*. We find that the activity of hCDK12/CyclinK toward a GST-yCTD substrate is independent of the proline-rich region C-terminal to the cyclin box of CyclinK and, unlike the activity of some CTD phosphatases (37, 38), is not stimulated by proline isomerization. Additionally we observe that CDK12 kinase and CDK9 kinase (P-TEFb) show identical sensitivities to the inhibitor DRB and similar sensitivities to the inhibitor flavopiridol (FVP); consequently, when either DRB or FVP is used to inhibit CDK9 *in vivo*, it is likely that CDK12 is also inhibited. Therefore, attributing a particular event to either one of these kinases based on inhibitor studies is not straightforward. We have also investigated the substrate specificities of full-length hCDK12 using GST-CTD fusion protein substrates with nominally 15 heptad repeats, which are all WT (YSPTSPS) or are all altered at one position (S2A, S2E, S5A, S5E, S7E). The WT and S7E substituted CTDs are phosphorylated to fairly similar extents by hCDK12/CyclinK, while the S2A, S2E, S5A, and S5E present themselves as weaker substrates (75%, 50%, 50%, and 25% activity respectively). These data argue that *in vitro* hCDK12/CyclinK, much like CTDK-I (16) and P-TEFb (39), is a promiscuous kinase that can phosphorylate multiple serine positions within the CTD (as an example, with the S2A substrate the complex must phosphorylate either the Ser-7 or Ser-5 position within the heptad repeats). Although our data agree with the previously reported preference toward a substrate prephosphorylated at the Ser-7 position of the CTD (11) as compared with that of Ser-2 or Ser-5, we do not observe this preference when comparing S7E to WT. The reason for this difference could be due to the nature of the substrate (our substrate is more reminiscent of the wild-type CTD in the number of heptad repeats (16 as compared with 3 in the other study), however the use of glutamate as a phosphomimetic is inferior to a true phospho-serine) or due to the use of a full length hCDK12/CyclinK complex. Additionally we have engineered, expressed, and purified an analog-sensitive form of hCDK12/CyclinK and shown it to be highly sensitive to the cell permeable, bulky adenine analog 1-NM-PP1.

To gain insight into potential non-catalytic roles of CDK12/CyclinK, we have identified proteins that co-immunoprecipitate with CDK12 from HeLa nuclear extracts under fairly stringent conditions. In view of the SR protein-like “arms” of CDK12 protein, it is perhaps not surprising that RNA processing factors are by far the most abundant functional class of CDK12-associating proteins. Over 30 splicing proteins are represented, including SRRM1 & 2, TRA2A & B, U2AF2, and SRSF1, 3, 4, 5, 6, 7, 8, 9, and 10. Also present are components of the PRPF19-CDC5L complex and a number of DDX and DHX helicases. While this report was in review another study of hCDK12 associated proteins, despite using somewhat different methods, identified a set of binding partners similar to these (40). In addition we find numerous snRNP proteins, especially those associated with U5, along with several subunits of SF3B. Beyond splicing, several 3′-end formation factors are also represented, including four poly(A)-binding proteins plus Pcf11 & CPSF1 and numerous subunits of the RNA exosome. Furthermore, the mass spectrometry identified PAP1L and PABP4; putative human homologues (by Uniprot Blast) of the yeast Ctk1-interacting protein GBP2 (26). Yeast contains only three proteins with canonical SR structures (RNA recognition motifs and an RS dipeptide-rich region): Gbp2, its paralog Hrb1, and Npl3. Intriguingly, all three of these proteins have been shown to interact with Ctk1 (26) suggesting an early evolutionary link between the Ser2 CTD kinases and the SR proteins. These results suggest that CDK12 is involved in multiple aspects of pre-mRNA processing, as is its yeast ortholog, Ctk1. In terms of splicing, we suggest that a number of SR proteins are recruited to the transcription elongation complex by virtue of RS domain interactions with CDK12. Thus we propose that CDK12 affects RNA processing events in two distinct ways: indirectly through generating factor-binding phospho-epitopes on the CTD of elongating RNAPII, and directly through binding to specific factors.

## Supplementary Material

Supplemental Data

## Footnotes

2 The abbreviations used are:

RNAPII: RNA polymerase II
CTD: C-terminal domain
FVP: flavopiridol
DRB: 5,6-dichlorobenzimidazole riboside
MCS: multiple cloning site.
