# Supplementary material for: Expression, Purification, and Identification of Associated Proteins of the Full-length hCDK12/CyclinK Complex
Source: J Biol Chem. 2014 Nov 26;290(3):1786–95. doi: 10.1074/jbc.M114.612226 (PMC4340420; doi:10.1074/jbc.M114.612226)
Supplement: Supplemental Data [file supp_290_3_1786__index.html]

Expression, Purification, and Identification of Associated Proteins of the Full Length hCDK12/CyclinK Complex — Expression, Purification, and Identification of Associated Proteins of the Full-length hCDK12/CyclinK Complex — Expression, Purification, and Associated Proteins of hCDK12 — Supplemental Data 

# Expression, Purification, and Identification of Associated Proteins of the Full-length hCDK12/CyclinK Complex

## Supplemental Data

**Files in this Data Supplement:**

- Supplemental File 1 (.xlsx, 67 KB) - List of hCDK12 associated proteins as identified by mass spectrometry.
